# Hürthle cell carcinoma: diagnostic and therapeutic implications

**DOI:** 10.1186/1477-7819-2-27

**Published:** 2004-08-11

**Authors:** Mohamed R Hanief, Laszlo Igali, Dimitrie Grama

**Affiliations:** 1 Imperial College London, Hammersmith Hospital, DuCane Road, London, W12 OHS, UK; 2 Department of Histopathology, Norfolk and Norwich University Hospital, Colney Lane, Norwich NR7 4UY, UK; 3 Department of Surgery, Sonderborg Central Hospital, 6400 Sonderborg, Denmark

## Abstract

**Background:**

Hürthle cell carcinoma is a variant of follicular cell carcinoma of thyroid. It may present as a low-grade tumour or as a more aggressive type. Prognosis depends upon the age of the patient, tumour size, extent of invasion and initial nodal or distant metastasis.

**Patient and methods:**

The case of Hürthle cell carcinoma is reported in a 79-year-old man who presented with a rapidly increasing lump on the left side of his neck, having had a right hemithyroidectomy for colloid goitre 24-years-ago. Fine needle aspiration cytology confirmed the presence of Hürthle cells, raising the possibility of a Hürthle cell neoplasm. The patient underwent staging and surgery. Histology showed Hürthle cell carcinoma and the patient underwent adjuvant therapy. The literature on Hürthle cell neoplasms is reviewed.

**Conclusions:**

Fine needle aspiration cytology may recognise Hürthle cell lesion but final diagnosis of carcinoma depends upon histological confirmation of vascular or capsular invasion. Staging and surgery in Hürthle cell carcinoma are similar to follicular carcinoma of thyroid with favourable outcome despite the controversy regarding the histological classification and adjuvant therapy. Elderly patients with Hürthle cell carcinoma need to be made aware of their poorer prognosis and should be offered more radical treatment.

## Background

The natural history of Hürthle cell carcinoma (HCC) is not well understood. It accounts for <5% of all differentiated thyroid malignancies. Hürthle cells are characterised by eosinophilic cytoplasm with trabecular/follicular growth pattern. [1]. Oncocytes are seen in follicular cell carcinoma but in HCC oncocytes represent more than 75% of cells, which exhibit a rather more trabecular growth pattern [2]. There is much debate regarding its clinical behaviour and little is known about the long-term survival of patients with HCC. Some studies have reported a relatively benign course while others have found the tumour to behave aggressively [3-6]. Most studies show that advanced age (>45), male sex, size of primary tumour (>4 cm), degree of invasion and recurrence are poor prognostic indicators [6-8]. Fine needle aspiration cytology is a good predictor of Hürthle cell neoplasm but is of little diagnostic value in evaluating HCC, since for a tumour to be deemed malignant one has to show vascular or capsular invasion [9]. Intraoperative frozen sections have a low predictive value. Udelsman *et al* found that in 96.4% cases with follicular neoplasm of thyroid, frozen section was neither informative nor cost-effective [10]. Well-encapsulated HCC run a favourable course while locally advanced HCC are associated with higher mortality and should be treated aggressively [4,11]. In a well-differentiated thyroid carcinoma death resulting from local disease is unusual and most die of distant metastases [12].

We report a case of a Hürthle cell carcinoma presenting in the left lobe of thyroid following a right hemithyroidectomy for a colloid goitre 24 years ago.

## Case presentation

A 79-year-old male was referred in March 2003 with a lump on the left side of his neck. The patient had noted a sudden increase in the size of the lump over the preceding two months. He did not report any neck pressure symptoms, weight loss or anorexia. His past history included right partial thyroidectomy for a solitary nodule (colloid goitre) in 1978 and repair of abdominal aortic aneurysm in 1994. He had suffered myocardial infarction in 1995 and had an episode of acute coronary insufficiency in January 2003. His recent coronary angiograms showed an occluded left anterior descending artery and echocardiogram revealed good left ventricular function. He was a non-smoker and consumed alcohol in moderation. He had been taking warfarin, diltiazem MR, lisinopril, uniphyllin, glyceryl trinitrate tablets and buccal suscard.

**Figure 1:**
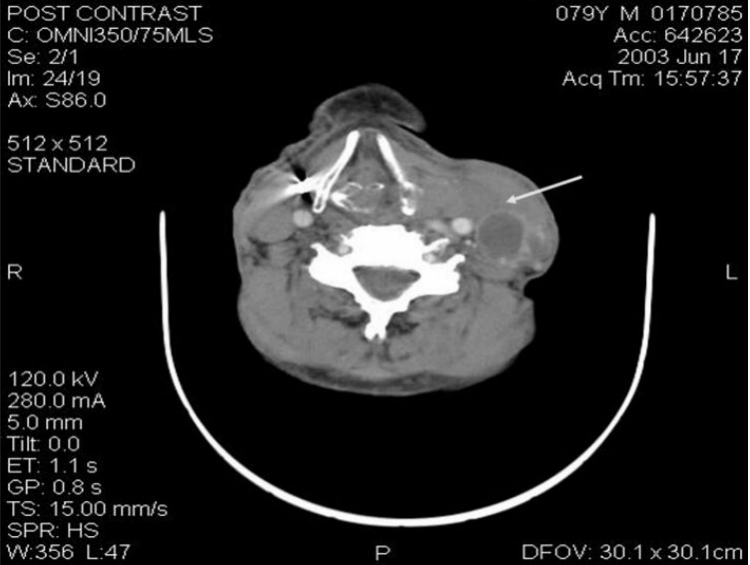
Superior extension of left goitre with 3 cm diameter complex mass deep to sternomastoid, posterior to carotid sheath. Note the displacement of larynx to the right.

**Figure 2:**
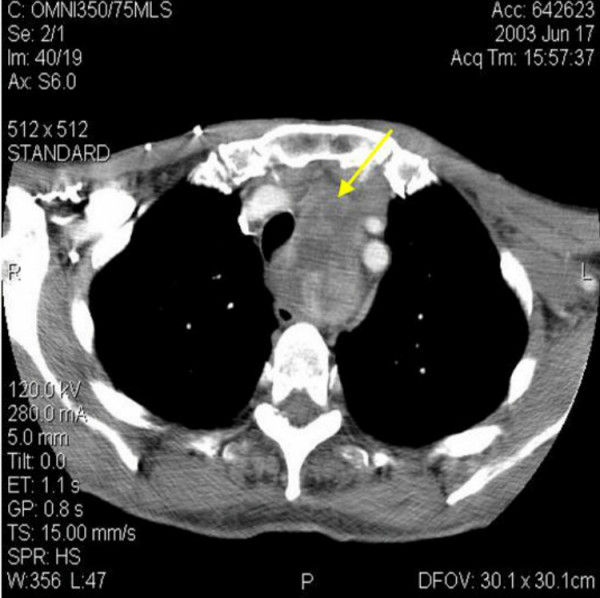
Mediastinal extension of left goitre.

On examination he had left sided goitre extending superiorly into the posterior triangle and inferiorly into the retrosternal space, with variable consistency. The trachea was deviated to the right and there was cervical lymphadenopathy on the left side. Systemic examination was unremarkable and fine needle aspiration of thyroid gland showed presence of Hürthle cells. Computerised tomographic (CT) scan with contrast enhancement (figure 1 &2) of the neck and thorax revealed large left sided thyroid goitre with significant mediastinal extension. It showed mixed attenuation with foci of calcification peripherally. There was a 3 cm complex mass on the left side of the neck, posterior to the carotid sheath structures and deep to the sternomastoid, indicative of lymph node metastases. Thyroid profile and routine blood investigations were unremarkable.

**Figure 3:**
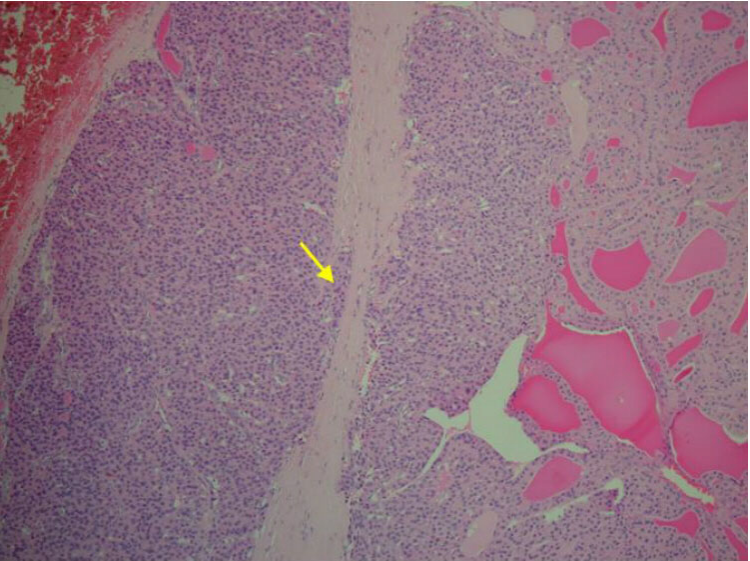
Photomicrograph showing capsular invasion (Haematoxylin and Eosin ×200)

**Figure 4:**
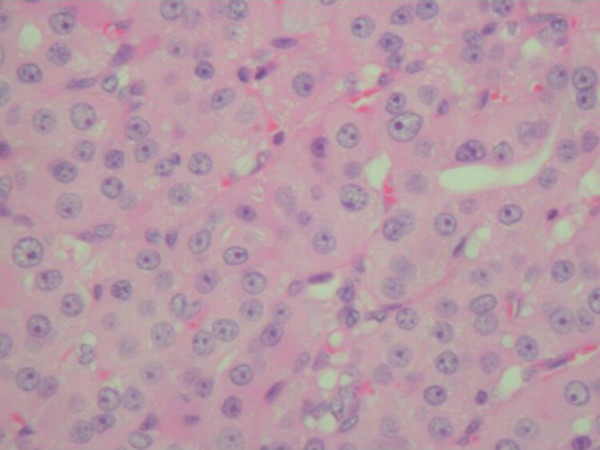
photomicrograph showing Hürthle cell note the eosinophilic cytoplasm and prominent nucleoli (Haematoxylin and Eosin ×500).

Based on the above findings radical surgery was planned. On exploration of the neck we confirmed left goitre with intrathoracic extension and enlarged lymph nodes under the sternocleidomastoid close to the jugulodigastric muscle and surrounding the carotid sheath. There was no remnant thyroid tissue seen on the right side following the previous thyroid surgery. Left hemithyroidectomy with modified neck dissection (lymphadenectomy, preserving all vessels and nerves) was performed. Macroscopic examination of the thyroid lobe showed a well defined solid pale brown mass approximately 8 cm in maximum dimension, surrounded by a narrow rim of preserved thyroid tissue. The lymph node specimen comprised of several nodules of partly necrotic tissue. Microscopic examination showed the thyroid lobe containing a Hürthle cell neoplasm, which was mostly encapsulated, with foci of capsular and vascular invasion. The two lymph nodes revealed metastatic Hürthle cell carcinoma. [pT3, N1a, Mx], (Figure 3 &4).

The patient had adjuvant therapy with oral radioiodine 131 (3060 MBq Sodium Iodine). He was put on a daily dose of 100 mcg of thyroxine. This was to be followed by a second dose of 5911 MBq of radioactive iodine six months from the time of the first dose.

## Discussion

Hürthle cell carcinomas are heterogeneous neoplasms that display a wide range of biological behaviour and accounts for less than 5% of all differentiated thyroid malignancies. The term HCC should be restricted to tumours with more than 75% of oncocytic cells [2]. Oncocytes are seen in follicular thyroid cell carcinoma and in papillary thyroid cell carcinoma [13,14]. On one hand patients with HCC live for years with slow growing tumour and lymphatic metastases and on the other hand, patients die of highly aggressive tumour with haematogenous spread.

Our patient had several indicators for poor prognosis such as his advanced age, male gender, large tumour size (8 cm), extra thyroid extension and nodal metastasis. Interestingly enough the patient had no pressure symptoms despite marked deviation of larynx, trachea and oesophagus, which may be due to previous right hemithyroidectomy. In elderly patients with sudden enlargement of neck mass and pre-existing thyroid conditions such as benign thyroid nodule, goitre (as in our case), Grave's disease or differentiated thyroid carcinoma, one has to bear in mind anaplastic thyroid carcinoma (ATC). In ATC local compression symptoms such as hoarseness, strider, dyspnoea and dysphagia occur as a rule [15-17]. In aggressive type of HCC haematogenous spread has been noted, but in ATC, at presentation patients are quite likely to have distant metastases involving lung, bone, brain and soft tissues [15,16].

Our patient had undergone fine needle aspiration cytology, which revealed Hürthle cells. Since the lesion was rapidly growing with mediastinal extension and nodal involvement, the patient underwent staging and left hemithyroidectomy with modified neck dissection. Histology confirmed HCC based on vascular and capsular invasion. Intraoperative frozen sections have low predictive value and are particularly not a sensitive test for diagnosing HCC therefore this was not carried out [10]. McIvor et al have clearly shown that FNAC can easily recognise the tumour as Hürthle cell lesion [9]. Cases with suspicious histology and over 50 years of age carry a high risk of cancer [18].

In the management of HCC the primary mode of treatment is surgical, ranging from hemithyroidectomy to total thyroidectomy. Larger tumours (>T2) require total thyroidectomy and lymphadenectomy if lymph nodes are involved [8]. Adjuvant radioiodine treatment or external beam radiotherapy is used for widely invasive carcinoma or locally advanced disease [8].

Several reports in literature have identified contra lateral foci of carcinoma in 40–70% of cases of HCC [11,19]. HCC is less responsive to radioactive iodine therapy [20] and taking into account the aggressive behaviour, it has been suggested that every Hürthle cell tumour greater than 2 cm should be treated by total thyroidectomy [21]. In 1990 they showed that recurrent disease was noted in 17% of patients treated with total thyroidectomy compared to 59% in cases where a more limited procedure was carried out [21,22]. Other authors support the role of total thyroidectomy as there is 15 to 35% incidence of multiple foci in HCC [23].

There are several reasons favouring the use of ^131^I remnant ablation after near-total thyroidectomy [24]. First, presence of thyroid remnant can obscure ^131^I uptake in cervical or lung metastases [25,26]. Second, distant (lung) metastases may be seen only on the post treatment whole body scan after remnant ablation [27]. Finally, remnant ablation may destroy residual normal follicular cells, which may become malignant [28] and any occult cancer that may recur years later.

**Figure 5:**
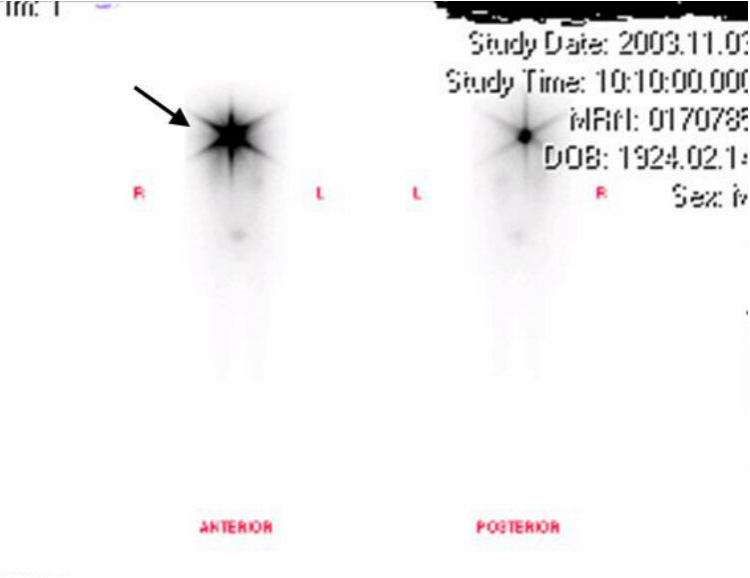
Whole body scan on November 3, 2003 following ^131^I ablation therapy on 28^th ^October 2003, with 3060 MBq Sodium Iodine (^131^I). Increased uptake is seen in the region of the thyroid bed. No abnormal accumulation was noted elsewhere.

**Figure 6:**
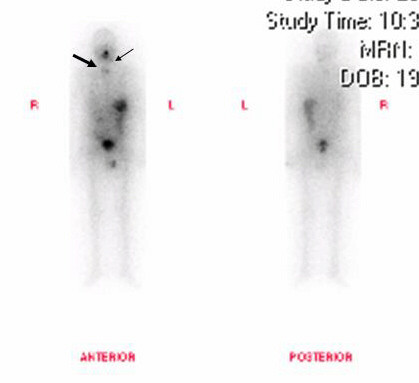
Whole body scan on 19^th ^April 2004 following ^131^I ablation therapy on 13^th ^April 2004 with 5911 MBq Sodium Iodine (^131^I). Two small focal area of uptake are seen in the thyroid bed. Low uptake focal area in the left lateral aspect of the neck, could possibly represent activity in a cervical node.

Radioiodine therapy has no overall effect on mortality but subgroup analysis has shown that those patients who receive radioactive iodine for adjuvant ablation of remnant thyroid tissue have lower mortality rate compared with patients who either did not receive treatment or in whom the indication was the presence of residual disease [29]. Radioiodine uptake in the elderly is much lower. Schlumberger and colleagues noted ^131^I uptake at metastatic sites in only 53% of patients over 40 years of age, compared to 90% in patients below the age of 40 [30]. Univariate analysis indicated that older age and large tumour size predicted worse survival rates due to aggressive nature of the tumour (extra glandular invasion and multifocal disease). One recent series reviewed medical records of patients between the years 1944 and 1995. Of the 89 HCC cases studied, 29% had only undergone lobectomy as initial treatment and 50% had undergone partial resection. Of the three quarters of the patients in this series who received radioactive iodine, only 38% of patients with known metastases showed positive uptake [29]. Another study clearly suggested that treatment with ^131^I to ablate the thyroid remnant and to treat residual disease were independent prognostic variables that favourably influenced recurrence, distant recurrence, and cancer death rates [24]. Our patient received radioactive iodine treatment in the postoperative period. He has been followed up with whole body scans (Fig 5 and 6), which indicate his response to adjuvant radioactive iodine therapy. He is on 125 mcg thyroxine in order to maintain a TSH level of less than 0.01 mIU/L and FT4 at the upper limit of normal (8–28 pmol/L).

Stojdinovic *et al* have treated 56 patients with HCC between the years 1940 and 2000 [8]. Of these patients 23(41%) had minimally invasive disease with no evidence of extra thyroid invasion (T2 N0 M0) and 33(56%) had widely invasive HCC. Primary mode of treatment was surgery ranging from lobectomy and isthumusectomy to total thyroidectomy with cervical lymphadenectomy in presence of lymph node involvement. Some patients received adjuvant radioiodine or external beam radiotherapy for widely invasive carcinoma. Study end points were relapse free survival and disease specific survival. They reported 8 years survival rate of 100% and 58% for low and high-risk cancers respectively. In their entire study cohort age was not found to predict the outcome but the most significant factor was widely invasive carcinoma.

Khafif *et al* in their series (42 patients with HCC between 1957–1997) used radioiodine in patients with distant metastases; none had thyroid remnant ablation with radioactive iodine [4]. They reported an overall survival rate of 90.5% and noted that age, size of tumour and extent of resection adversely affected the prognosis.

Hürthle cell lesion can be easily picked up on FNAC but to make a diagnosis of HCC one has to demonstrate vascular or capsular invasion. Intraoperative frozen sections have low predictive value and cases with advanced age (over 50), rapid enlargement of lump and palpable nodes should be regarded with high index of suspicion for presence of HCC. HCC or other differentiated carcinomas of thyroid in the elderly patients are generally more aggressive with less favourable prognosis compared to younger patients. They should be offered total thyroidectomy and selective lymph node dissection (when lymph nodes are involved) followed by ablative radioiodine therapy, provided they can withstand the above treatment. Coexisting medical disorders should be recognized and managed effectively prior to surgery [31]. Further research is needed to clarify the role of adjuvant radioiodine therapy in the management of HCC.

## Competing interests

None declared.

## Authors' contribution

**MRH **managed the patient, searched the literature and drafted the manuscript

**LI: **did the histological study, and contributed to pathological aspects in the present study

**DG: **Managed the patient, conceptualise the present report, edited the manuscript and coordinated after reviewing the manuscript
